# The lived experience of remembering a ‘good’ interview: Micro-phenomenology applied to itself

**DOI:** 10.1007/s11097-022-09844-4

**Published:** 2022-09-10

**Authors:** Katrin Heimann, Hanne Bess Boelsbjerg, Chris Allen, Martijn van Beek, Christian Suhr, Annika Lübbert, Claire Petitmengin

**Affiliations:** 1 Interacting Minds Center, Aarhus University, Aarhus, Denmark; 2 Max Planck Institute for Empirical Aesthetics, Frankfurt, Germany; 3 Department of Clinical Medicine, Aarhus University, Aarhus, Denmark; 4 Elective Surgery Center, Silkeborg Regional Hospital, Silkeborg, Denmark; 5 Cardiff University Brain Research Imaging Centre, Cardiff University, Wales, UK; 6 Department of Anthropology, Aarhus University, Aarhus, Denmark; 7 Department of Neurophysiology and Pathophysiology, University Medical Center Hamburg-Eppendorf, Hamburg, Germany; 8 Institut Mines-Télécom Business School, Paris, France; 9 Archives Husserl, École Normale Supérieure, Paris, France

**Keywords:** Micro-phenomenology, First person method, Second person method, Subjective experience, Relationality, Interview quality

## Abstract

Micro-phenomenology is an interview and analysis method for investigating subjective experience. As a research tool, it provides detailed descriptions of brief moments of any type of subjective experience and offers techniques for systematically comparing them. In this article, we use an auto-ethnographic approach to present and explore the method. The reader is invited to observe a dialogue between two authors that illustrates and comments on the planning, conducting and analysis of a pilot series of five micro-phenomenological interviews. All these interviews asked experienced researchers of micro-phenomenology to browse their memories to identify one successful and one challenging instance of working with micro-phenomenology. The interview then focused on this reflective task to investigate whether applying the method to itself might reveal quality criteria. The article starts by presenting a shortened and edited version of the first of these interviews. Keeping the dialogue format, we then outline the micro-phenomenological analysis procedure by demonstrating its application to part of this data and corresponding passages of other interviews. We focus on one unexpected finding: interviewed researchers judge the quality of an interview in part based on a connection or contact between interviewer and interviewee. We discuss these results in the context of the means and intentions of the method and suggest avenues for future research.

## Introduction

Micro-phenomenology is a research tool enabling the investigation of subjective experience. Comprising an interview and analysis technique, it allows for detailed descriptions of brief moments of subjective experience and provides protocols to compare them systematically. 

Micro-phenomenology has been developed by Claire Petitmengin within the context of the neuro-phenomenological research program as advocated by her PhD supervisor Francisco Varela. According to Varela, the study of the human mind, body and psyche cannot be accomplished by relying on exploring physiological activities alone (Varela, 1996). Instead, it requires a rigorous discipline to study human experience, to meet and enter into a dialogue with cognitive neuroscience. Micro-phenomenology developed from the "entretien d'explicitation", an interview method initially proposed developed by the French psychologist Pierre Vermersch for educational purposes and for analysing professional practice (Depraz et al., 2003; Vermersch, 1994). To adapt Vermersch's interview technique to research within cognitive science, Petitmengin developed a method for analysing verbal reports and detecting possible regularities to enable derivation of generic structures that refer to the experience targeted by the interview and the cognitive (neuro-)science experiment.

Micro-phenomenology is now used to study lived experience across a range of domains. It has been applied as a stand-alone tool and combined with other measurements (for example, in neuro-phenomenology, see Lutz, 2002). It has been used in multiple areas, including in education, for exploring the emergence of a mathematical idea (Petitmengin, 2001), for fostering sustainable behaviour (Frank, et al., 2022), or for revisiting the phenomena of writer´s block (Horwitz et al., 2018). In clinical domains, examples include the investigation of anticipation of epileptic seizures (Petitmengin et al., 2006) or episodes of pain in fibromyalga (Valenzuela-Moguillansky, 2013). Also, in technology, for example, to illuminate and advance clothing design (Petreca, 2016), artistic, to explore musical experiences (Vion-Dury, 2014; Vásquez-Rosati, 2017), and contemplative domains (Petitmengin et al., 2017), to name only a few. In all these fields, the combination of interviews and analyses enabled mapping multiple dimensions of the experiences and made important contributions to the respective fields. Previous publications described the premises and principles of the micro-phenomenology interview (Petitmengin, 2006) and later the analysis technique (Petitmengin et al., 2018). However, micro-phenomenology is relatively young and still refining its theory and praxis.

Within this volume, the present article outlines micro-phenomenology as one of several methods to explore and document (others’) experience. We do so by showing how it is practised and experienced by researchers who use it. To achieve this, we were inspired by an article by Cann and DeMeulenaere (2012) that portrays the method of "critical co-constructed auto-ethnography" by literally demonstrating it: Their article is written as a dialogue between the two authors. Our dialogue, represented below, relies on actual interactions and preserves their basic style, content and progression but combines snippets and edits from conversations at different points in time to sharpen the arguments and achieve the best possible illustration of the process. The reader is invited into a dialogue between two authors as they develop the idea for a pilot series of micro-phenomenological interviews and then conduct and analyse those interviews. This should allow the reader to witness the conduct of an interview as-well-as subsequent steps of analysis and interpretation.

In the following, we start the dialogue format by presenting excerpts of a conversation that took place between Katrin Heimann (Kat) and Hanne Bess Boelsbjerg (Bess) in October 2020. Kat and Bess have been colleagues and close friends for six years. During this conversation, Kat's initial research question is: What makes for a good interview?

*Kat: Bess, thanks for being here with me today.*

*Bess: My pleasure. Shall we begin by recapping what we agreed to do today?*

*Kat: Yes. Our plan is for me to run an interview with you, specifically a micro-phenomenological interview, sticking closely to the guidelines stipulated by Claire Petitmengin in 2006. I decided to interview five experienced micro-phenomenology researchers about their experiences with the method. And you are the first*.

*Bess: Great. Let's get started*.

## The interview

### Introduction of interview technique to the interviewee

The micro-phenomenological interview differs substantially from everyday conversation and most interview types. Before starting an interview, it is important to introduce the objectives and principles, to adjust expectations, and help interviewees focus on describing their experience. The following dialogue illustrates such an introduction. If time allows, especially for interviewees new to micro-phenomenology, we recommend that such an introduction be complemented by a separate training interview on an unrelated experience to allow the interviewee to get used to the particular style (not illustrated here).

*Kat: Let's begin, as one usually does, by introducing the method.*

*Bess: Of course!*

*Kat: Ok–today, we will run a micro-phenomenological interview together. The micro-phenomenological interview technique is designed to help a person increase awareness of their subjective experience and describe it in the interview. By ‘subjective experience’, I mean any events and states you live through which are subjective or ‘private’, in the sense that most observations via another person, and even physiological measurements or similar, are limited in what they can reveal about them. This might comprise anything from sensations and feelings to mental images, memories, thoughts and much more.*

*Bess: I guess some interviewees might ask here: why is it necessary to increase the awareness of my own subjective experience? If it is my experience – how come I might not be fully aware of it?*

*Kat: It is a premise of the method that reporting one's own lived experience is not simple. This is partly because the experience might be hard to put into words and partly because, in our daily life, we tend to focus our attention only on parts of our experience.*

*Bess: And what is it that we do and do not focus on, usually?*

*Kat: In ordinary situations – as-well-as conversations about our experience–we mostly dedicate our attention to the content or ‘what’ of experience, often followed by judgements and explanations of it. For example, when we realise we remember something, we likely just focus on the content of that memory–which object, person or event comes up in the memory. And maybe ask ourselves: why is that important now?*^1^* It is unusual for us to notice ‘how’ precisely we experience something. For example, how we remember the respective object or person or event: Do I ‘see’ them in front of my inner eye? Do I hear something? Does the memory involve a bodily sensation? And if it includes an image: is it a focused or blurry image, still or moving, black and white or in colour, or specific in any other way? Micro-phenomenology is designed to aid this focus on the ‘how’, enabling us to become aware of what might often go unnoticed or unreported and report it to another.*

*Bess: Right. And how is this done? What should I expect as an interviewee?*

*Kat: In a micro-phenomenological interview, I first ask you to describe how you lived a specific moment. For example, I might ask you about what happened when you just remembered somebody – say your grandparents. That is, I’m interested in this specific instance of remembering them–not how you remember them in general. Which precise moment we choose as a "target experience", depends on the question we are interested in and our experimental design. Still, invariably, we do not select a type of experience, but a single occasion, a concrete and specific moment to explore that is ideally defined by a discrete beginning and end.*

*Bess: Ok, so you ask me to remember a specific moment?*

*Kat: Yes, as the experiences explored are always described in hindsight, every interview starts with what we call an invitation to evocation: This means I will invite you to go back to the beginning of the moment that we want to explore. In essence, "evoking" such moments is much like the common experience of trying to place ourselves back to the moment in which we, for example, the last time saw our misplaced car keys. And, as an interviewer, I will help you in this endeavour by asking you to take the time to remember it in as much detail as possible. So, thinking about the keys to be found, I might ask you: When did you last have them? Where precisely were you? Which bodily position were you in? What could you see, hear, feel or sense in that very moment? Thus, starting an interview, I will ask you to do something similar. So, for example, I will invite you to go back to the moment just before you remembered your grandparents. Only once this moment has become sufficiently present to you, or evoked, then I ‘ll ask you to walk me through the course of the experience following this moment, as you remember it.*

*Bess: And will you tell me what interests you about this moment?*

*Kat: No. Importantly, I will not pre-structure this initial report by asking pre-determined questions. Rather, my role is to encourage and support you in simply reporting anything that happened to you from the start to the end of our defined target experience, independently of whether it might relate to my research question or not. The aim is to gain a report about a specified experience that is as complete as possible.*

*Bess: And what does "supporting me" in this report entail?*

*Kat: After guiding you in the evocation by helping you to recall the context of the experience, I might first try to lead your attention to the temporal unfolding of your experience with open questions not suggesting any specific content, such as "How did you start?" or "What happened then?" or "Did anything else happen?".*

*When, in your report, we have moved through the entire experience once, I will then try to report your description back to you. I will be careful to use your expressions rather than my interpretation. I might even repeat parts of your description word-by-word, including imitating your intonation and gestures. This repetition attempt should allow you to reexamine your report and possibly correct me, or even your own words, to better describe your experience. Re-listening to your words might also intensify the evocation, allowing more details of the experience to come into focus. Either way, I would like you to interrupt me to correct or complement your report. Does that sound ok?*

*Bess: I will try to do so! But just to be clear: This first report is about gathering an overview of the temporal unfolding of the experience, right? So, we are kind of like establishing a timeline.*

*Kat: Indeed. Though interviewees might also report about events or states co-occurring or overlapping with each other. Now, when we have checked and refined your initial report in this way, I will start to examine its single moments in more detail. For this, I will guide your attention to a moment in your report and might suggest and assist you in evoking this particular time. For example, I might say: So, you just said, the first thing that happened was that you remembered your grandmother, your mother's mother, to be precise. Could you go back to the very beginning and tell me: how did this experience unfold. Just as before, I might then use other open questions to assess the temporal course, such as "What happened then?" or "What happened just before that?". And finally, I might also use "how" questions to further explore a certain aspect of your experience in all its non-temporal dimensions. For example, if you tell me: "Well, firstly, I recalled her hand". Then I might ask: "How did you recall her hand?".*

*Bess: You mean: "why" did I recall her hand in particular?*

*Kat: No, and it is really good to clarify that: I'm asking "how", not "why". "How" will invite you to describe what happened in your experience, whereas "why" would lead you to speculate as to the reasons for your action, which is not what we are interested in.*

*Bess: I can imagine that expressing this "how" of my experience is not always easy. What if I neither really saw, nor felt her—but still remembered her somehow?*

*Kat: Indeed, it is at this point that we may brush up against our limitations of verbally articulating lived experience. It can be difficult to describe some experiences with words. If this should happen in the interview, just express the struggle. Sometimes, it just needs some time to let it become clearer. Sometimes, further questions might help, but we also need to be careful not to encourage you to describe elements that were not part of your original experience and to express doubt when you're not sure. Doubt is an important clue about authenticity and being at the limits of your memory report.*

*Bess: Right. Anything else?*

*Kat: Yes, lastly, but very importantly, sometimes you can articulate detail of your experience, but for some reason, you might not feel comfortable sharing it. In this case, even though we know each other well, please signal this, and we will either skip this part or, if you wish, stop the interview altogether. This is what we call the "privacy contract", which I will reiterate during the interview by frequently asking you: "If you agree, could you…".*^2^

*Bess: I agree.*

#### Exemplification of experimental design and interview process

This sub-section exemplifies the conduct of one interview aimed at a single experience. There are two main ways of defining a target experience for an interview. One possibility is to help the interviewee re-evoke a specific experience they lived through prior to the interview situation. Such setup allows us to maximise ecological validity but presents challenges due to weakened memory traces that refer to events that can be some time in the past.

An alternative is to define a target experience immediately before an interview. This might involve a task, game or any other experience which is then targeted by the investigation. This can provide fresh target experiences with a clearly defined start and end. It also allows for combining micro-phenomenology with other methods such as neuroimaging, i.e. neurophenomenology. Lastly, it allows the management of the duration of the experience. Durations that can reasonably be explored in one session of a micro-phenomenological interview are relatively short, ideally, a few seconds, where it is rarely feasible to target experiences lasting more than a few minutes. The following dialogue introduces the design of this project and lets the reader observe an interview.

*Kat: In the past, I've had numerous conversations with other micro-phenomenology researchers about successes and challenges when applying the method–and in particular, the interview. In our conversations to prepare this article, I began to wonder: are there criteria for a 'good' interview that could be revealed by applying the method to itself? This is what these pilot interviews aim to investigate.*

*Bess: And what experience do you think would allow us to explore this question?*

*Kat: My very first thought was that I would simply help you to evoke the experience of a good interview and explore it. However, such a target experience might lie very far back in time and might stretch across several hours, which is an experience not easy to explore with micro-phenomenology. Therefore, a little twist that might allow me to get to the question would be to ask you to recall and 'good' and a 'challenging' experience you've had with micro-phenomenology and then interview you on the process of the recall and selection, rather than on the original experience itself. Would you be up for that?*

*Bess: Yes, let's try that!*

*Kat: Ok, then I invite you now to take a minute to go through your previous experiences with micro-phenomenology – it could be as an interviewer or as an interviewee. More specifically, I would like you to identify two memories: one that you consider representative of a successful interview, and one that you found challenging. Take your time and let me know when you are there.*

*Bess: [sits in silence for ~ 40 s, eyes closed]: Ok, I am here.*

*Kat: I would now like to explore this process you just went through, the process of selecting those moments.*^3^

*Bess: Yes.*

*Kat: Then, if you agree, I invite you to go back to the moment, just a minute  ago, in which you started revisiting  your experiences. Take a second to recall this beginning as vividly as possible, maybe identifying what precisely I said to you, adopting the same position as when you were listening to me, try to re-experience anything else that happened around that moment.*^4^* When you feel you are there, please start with an overview – what did you experience from the start until you told me, "Ok, I am here"?*

*Bess: Yes. [Closes her eyes again and starts to speak. Kat is looking at Bess as well as at the notes Kat is writing].*^5^* So, I started a bit earlier while you were still talking because you had already mentioned yesterday that I would be asked to remember my previous interview experiences.*^6^* I first browsed my memory quite openly, thinking of some of the recent interviews I had conducted. And quite quickly, I came across the positive one. It is a very resourceful interview–I myself have used it in a presentation. So, it was easy to pick; it came by itself, in a way, immediately narrowing down the search.*

*Um… and the other one… that was a bit difficult. I first browsed situations of trying to use micro-phenomenology within other contexts – where I was not really bold enough or felt that it wasn't welcome. So, I thought those instances didn't fit this purpose. Then I looked for a real micro-phenomenology interview, and finally this appeared: an interview in which there was a moment of something that felt restraining, difficult, and that I needed to address after the interview. [Stops talking and opens her eyes].*

*Kat: Thank you! I will now repeat what I understood and please interrupt me when you want to correct or add something: So, you said you started the browsing a bit earlier, since I had already mentioned that the task would be about remembering previous interview experiences. So, you thought of some of the recent interviews you did and you quickly came across the positive one.*^7^

*Bess: Yes, that's right.*

*Kat: You mentioned it being a very resourceful interview, one that you have used before in a presentation. Could you describe: How did you experience this aspect in this moment, this "it being resourceful and used in a presentation afterwards"?*^8^

*Bess: Oh, um [short break]. So, the memory that came up was good. It was hmmm [sound expressing satisfaction], a good interview. About the presentation… I think that came a bit later, as an additional justification. I am not totally sure.*^9^

*Kat: Thank you for that clarification. So, this interview popped up quickly, it came by itself, seemingly, as a good memory, a good interview, it had this "hmmm". Then, the challenging experience…*

*Bess: [interrupts] Oh sorry, I just realised that I confirmed this later, after I had selected a positive and a negative experience. I felt I still had some time after the selection because I had started before you even asked the question. Therefore, I concentrated again and went back to the two choices I had made beforehand and asked myself: "What was it that I experienced as something that was good, or felt easy, in flow or valuable?" And for the other one, I tried to identify the struggle I had, going back to the interview and considering: "Was it around here or there?" And when I found out, I thought, "Ok, these choices are good enough". And then I told you, "I am here".*^10^

*Kat: Right! Thank you for that correction. So, you had selected two interviewer experiences. But you still had time, so you went back to the two choices you had made and asked yourself what it was that you experienced as good or valuable or – with respect to the negative one – as a struggle. And when you arrived at an answer to that, you thought, "Ok, these choices are good enough". And then you told me, "I am here".*

*Bess: Yes.*

*Kat: If you agree then, I would like to go back to this later phase, in which you went back to the two choices and asked yourself on what basis you had selected them. From there we'll go into more detail.*^11^

*Bess: Sure.*

*Kat: Would you agree then to take some time to let this specific moment come back, the one in which you go back to the first choice, the good interview: how precisely did that happen, how did you do this?*^12^

*Bess: Yes. What precisely am I doing there? … [Short break] I tried to recall the interview. So instead of something just popping up when I got this condensed feeling of hmmm, it was more like voluntarily entering the memory of the interview along a timeline, browsing through the different moments in it. And… so there was this strong sensation at the end of the interview. And one in the beginning as well. I really felt like I connected to Leonore there.*^13^* And I was like, "Ok, that is part of why I decided in favour of this interview."*

*Kat: Ok, thanks! So, you were entering the experience of the interview in a timeline, browsing its moments. And you encountered a strong sensation of connection at the end and also at the beginning of the interview. This was part of why you decided upon this interview. Would you agree to go back to this moment where you first sensed this connection and describe what happened there, step-by-step?*

*Bess: Yes… During the interview, Leonore told me about how she went back to her home island when her grandmother died. In this memory, she plucks a flower that symbolises her grandmother and travels with it over the island. So, in my imagination I see the flower.*^14^* Then I start to see Leonore walking.*

*Kat: So, in your imagination you see the flower. And then you start to see her walking. May I ask you: When you start seeing her walking: what happened? What was that experience like?*^15^

*Bess: That would be like: I see her walking with her back in front of my eyes. Some of it is very visual, the sense of following her along, watching her, visually in her descriptions or in the things that I conceive of as her descriptions. And the other sense of it is more bodily, a sensation that goes on in the upper and lower stomach, something in the forefront of my body.*^16^* Something to hold on to or grasp into. It comes with a sensation of being warm. A way of feeling like supporting another person. I don't know how to exactly express it. Um… it's a way of being present myself and leaning into the situation with a sort of strength or saying: "you can trust me"… or maybe "you just go ahead with that"… So that is the sense of connecting. I'm not identifying directly with her. It's more that I lend her my presence. And that's bodily based.*

*Kat: You recall a bodily based sense of connection, lending her your presence. And that's bodily based. If you agree, could you go to this moment and describe how this lending is bodily based? ….*

This transcript presents a short, edited excerpt of the full interview. Kat and Bess explored many moments and simultaneous characteristics of the experience, which took about three and a half hours before Kat and Bess agreed to conclude the exploration.

At that point in time, Bess voiced that it was getting hard for her to evoke specific moments. Data retrieved under such circumstances, whether caused by fatigue or other factors, are considered less reliable, which is why, in this particular case, Kat decided to end the interview. According to the method, this is done by the interviewer repeating the experiential report once more, and asking the interviewee if there is something, anything, they would like to add. If not, the interviewer gently asks the interviewee to return to the present situation, away from the evoked one.

Over the following two weeks, Kat conducted four additional interviews using the same protocol with Martijn van Beek, Chris Allen, Christian Suhr and Claire Petitmengin, all of whom were also involved in the refinement of the analysis^17^ and the formulation of this manuscript. Among others, we found that all five participants reported judging the interview quality mainly based on the sense of connection or contact experienced between interviewer and interviewee. Rapport between interviewer and interviewee is already understood to play an important role in qualitative research (e.g. Gilligan, 1982). However, we decided that the prevalence of this theme across interviews motivated it as the focus of the above excerpt and analysis presented. We now turn to this analysis.

## The analysis

Micro-phenomenological analysis allows us to uncover the structure of each individual experience (specific analysis) and, based on these, to then derive the generics of the experience type across interviewees (generic analysis)^18^. The detection of each of these structures depends on preprocessing steps, described in the following sub-section, before we outline the derivation of structures describing the experience.

### Processing the transcripts

The preprocessing consists of three steps: Firstly, the interview is transcribed following the guidelines given in Valenzuela-Moguillansky and Vásquez-Rosati (2019). This includes notations of non- and para-verbal speech elements, including breaks, stresses, gestures and more, depending on what is needed to answer your research question. Then, only the experiential data is identified and extracted, which involves deselecting all contextual information, comments, or reflections that did not describe aspects of the target experience itself (satellites). At the same time, one should mark or remove all other parts of the report that seem less reliable, that is where the interviewee expressed doubt, or the phrasing of the interviewer is likely to have primed the response. Lastly, the remaining informative verbatim excerpts from the transcript, or ‘descriptemes’, are re-organised so that the order of the quotes corresponds to the order of events in the target experience.

*Bess: So, given the transcript of our verbal interactions, would you illustrate the next steps of preprocessing?*

*Kat: Sure. Let's start with the identification of experiential data.*

*For example, consider your utterance, "It is a very resourceful interview, I have also used it in a presentation afterwards". While you had initially used this sentence to describe the interview you identified as successful, during our interview, we concluded that you did not think about this presentation in the moment of interest, but it likely occurred during the interview.*

*Bess: Meaning, you regarded it as a satellite of the target experience and removed it from our pool of experiential descriptemes.*

*Kat: Yes, at this stage of the process we would also discard any elements where I, the interviewer, might have led you, especially where this might open the door to confabulation, as we'll discuss later.*

*Bess: And this possibility should be kept in mind when analysing the data.*

*Kat: Precisely. Depending on your research question you might also discard quotes on the basis of relevance to the target.*

*Bess: This leaves us with the step of reordering the descriptemes.*

*Kat. Yes, usually, an interview proceeds in loops: first, we establish a rough timeline of the entire experience, then we explore single moments in more detail, meaning that descriptions of the same moment are not always given consecutively but are scattered throughout the transcript. Regrouping the quotes in order gives us the basic temporal succession or ‘diachrony’ of the experience, as we call it. For example, in one of your early outlines of the target experience, you said, "I concentrated again and went back to the two choices I had made beforehand …". At a later stage of the interview, you refined this statement saying, "…it was more like voluntarily entering the interview along a timeline, browsing through the different moments". Thus, in the reordered sequence of experiential reports, these two quotes would be arranged after one another, so that when reading through the rearranged quotes one would follow the unfolding of the experience rather than the interview.*

*Bess: And this is essential for uncovering the specific structure of the experience in the next step.*

#### Derivation of specific structures

Micro-phenomenology allows for extremely rich experiential reports of short moments in time. Comparing such experiences across trials, individuals, or conditions requires operations of abstractions that turn the vast amounts of details assessed into manageable numbers of structural dimensions. Specific structures are the results of such abstractions, reflecting the dimensions of a single experience. We distinguish between two kinds of structure: Diachronic structures map the unfolding of an experience over time, portraying it as a succession of moments or states and their potential simultaneity. In contrast, synchronic structures sketch the network of descriptive categories or dimensions present in a single moment of the unfolding experience. The following dialogue gives examples of each kind of structure and how they are derived from Bess's report.

*Bess: The process of analysing the specific structures usually starts on the diachronic level, mapping the consecutive moments of an experience as it unfolds over time.*

**Fig.1:**
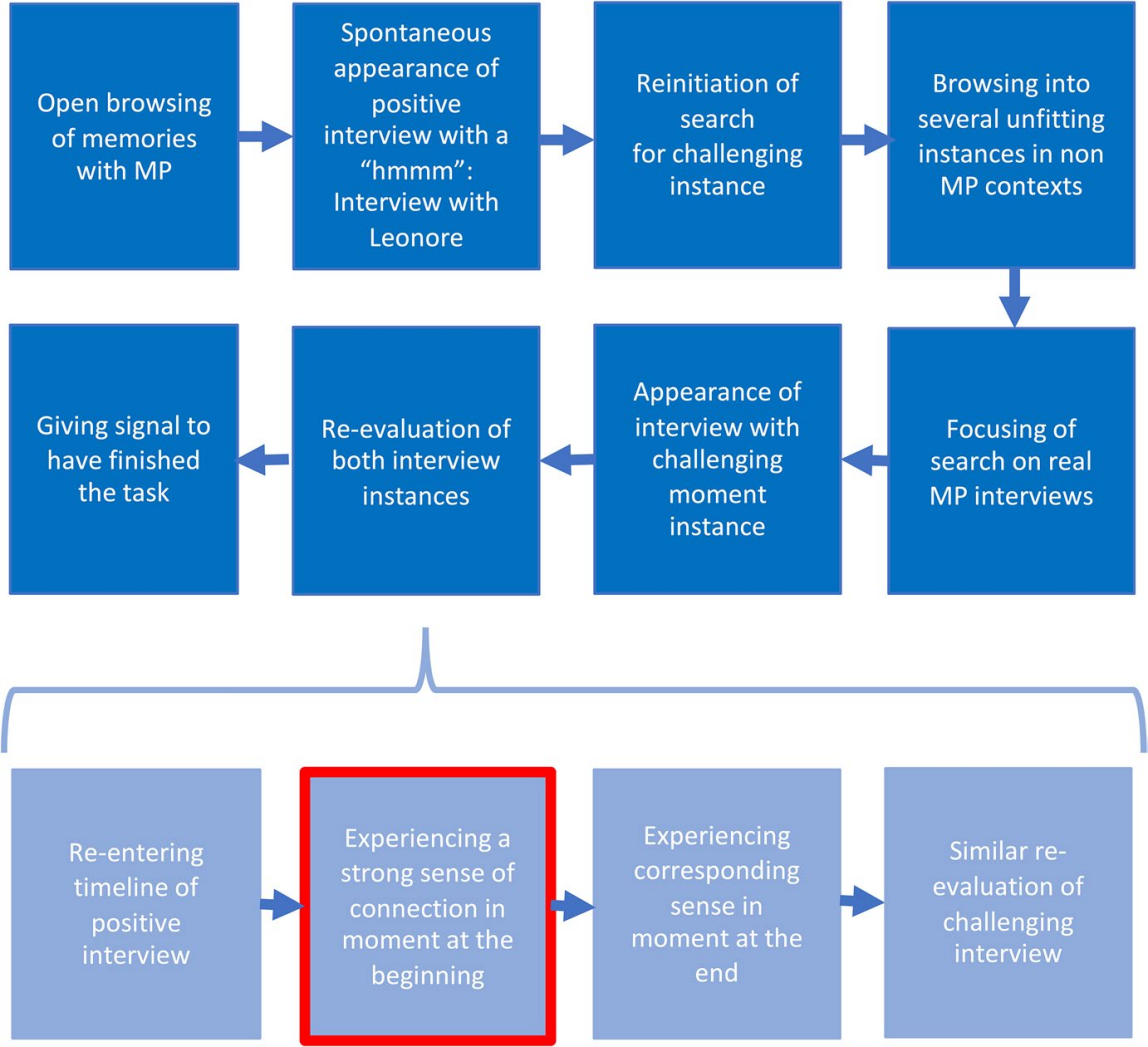
Specific diachrony of Bess's experience of the experimental task. Framed in red is the sub-moment analysed further below

*Kat: Yes, already, the preprocessing step of reordering the quotes affords to roughly group the quotes into seemingly separable moments. To arrive at the specific diachronic structure basically means clarifying this grouping and giving each resulting moment a title that describes what is happening in it. To give an example, I separated the reordered quotes of our interview into the following rough phases: The first quotes describe the initiation of the search while I was still formulating the question, a kind of ‘open browsing’. The next moment you describe is how, as a result of this activity, you spontaneously come across a positive interview, the one having this "hmmmm". Having found this, you re-engage into searching for the more challenging instance, browsing into several situations that seem outside the context. You thus refine your search further and finally come across an interview that includes a difficult moment. But your experience does not stop here. Rather, you then go back to both choices in more detail, trying to identify the precise moments or aspects that caused you to classify them as a positive or challenging instance. For the positive instance, you went on to specify that you did so by entering the experience in a timeline, browsing its moments. And for two of those moments, you encountered a strong sense of connection that validated your choice, which finally allowed you told me that you were done. I have illustrated this specific diachrony in *Fig. 1*:*

*Bess: And for each of these phases, it is possible to derive a synchronic structure describing how this moment was experienced. That is the network of descriptive categories characterising this moment.*

*Kat: Yes. Such categories are generated by performing a series of abstraction operations that consolidate quotes referring to raw experience into broader, more general categories (*Petitmengin et al., 2018*). For example, let's go back to the descriptemes concerning the "sense of connecting" that you reported experiencing when re-evaluating the interview that appeared to you as successful.*

*"So, in my imagination I see the flower. Then I start to see her walking. That would be like: I see her walk, having her back in front of my eyes. Some of it is very visual, the sense of following her along, watching her, visually in her descriptions or in the things that I conceive of as her descriptions. And the other sense of it is more bodily, a sensation that goes on in the upper stomach and lower stomach, something in the forefront of my body.”*

*Bess: To map out the synchronic structure of this moment, we are looking for potential ‘structural statements’ in these quotes – those that point to or allow abstraction of a distinct dimension that captures how this moment was experienced – dimensions that might reveal the essential to a type of experience.*

*Kat: Indeed, to start with, we have "a sensation that goes on in the upper stomach and lower stomach, something in the forefront of my body”. So, a structural feature of the sensation is a specific bodily location.*

*Bess: Previously, you mentioned that we are dealing with abstract operations here. There are a few of them used in micro-phenomenological analysis, aren’t there?*

**Fig. 2:**
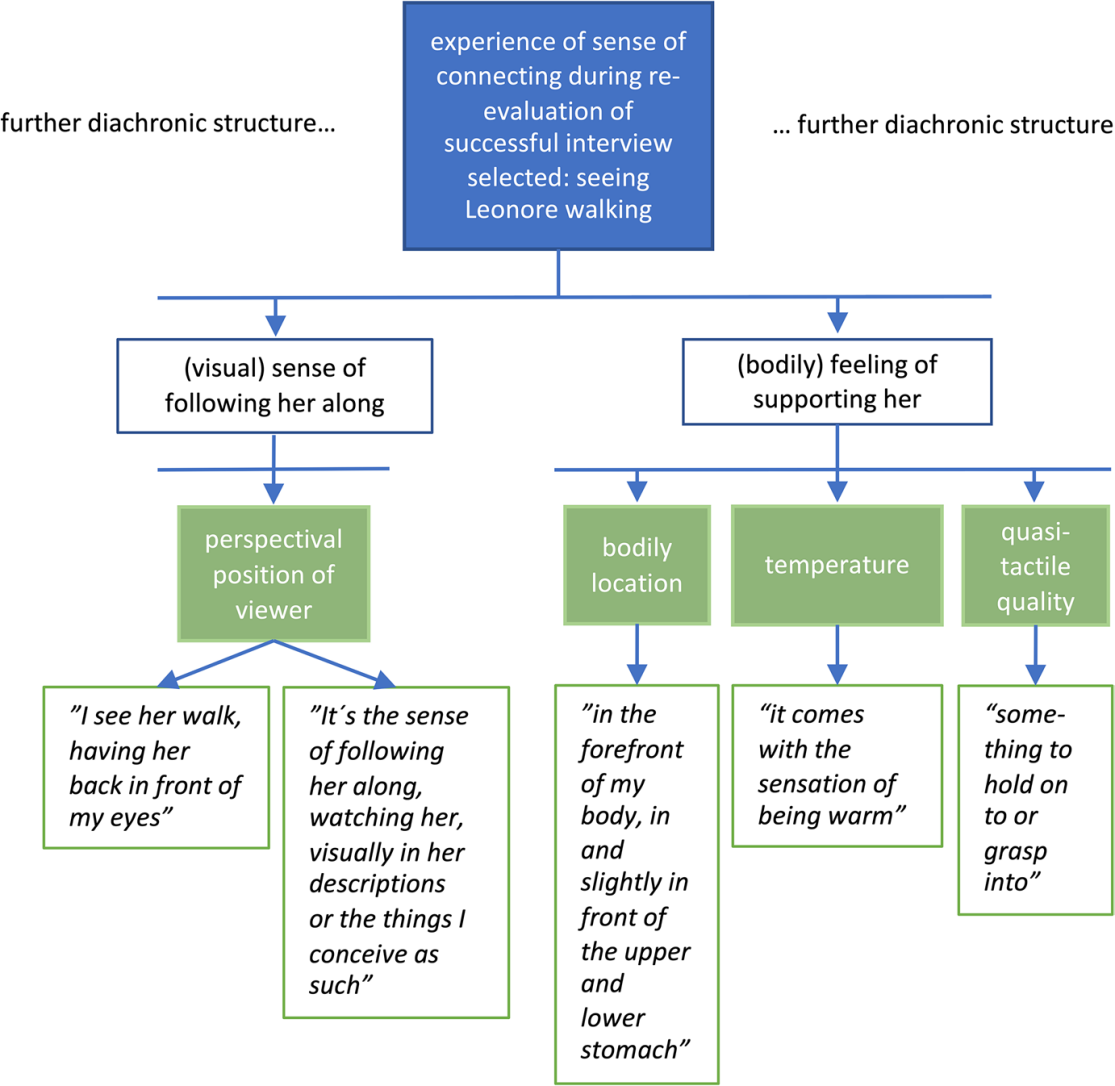
The specific synchronic structure developed for the ""sense of connecting"" that Bess experiences when re-evaluating the interview that she selected

*Kat: Yes, this derivation belongs to the type of synchronic called ‘classification’: I classified the descripteme as an instance or instantiation of the synchronic category: "bodily location".*^19^* Likewise, when you said, “I see her walk, having her back in front of my eyes”, this is a visual instantiation where you gave a “perceptual position of view”. These operations are formalised in *Fig. 2*, and are, by convention, described by the arrows pointing from the synchronic category downwards to the instances or descripteme. In total, there are three couples of abstract operations in micro-phenomenological analysis: 1) classification and instantiation, 2) aggregation and fragmentation and 3) generalisation and specialisation.*

*Bess: The figure also shows that you grouped bodily location, temperature and quasi-tactile quality under the broader category “(bodily feeling) of supporting her” and these relations use a different type of arrow.*

*Kat: Yes, we considered them components or fragments of this more abstract category, thus using an operation of aggregation, expressed by down-pointing but rectangular arrows in *Fig. 2*.*

*Bess: And so you proceeded with these operations until you had included all important descriptemes as instances of some categories. And you did this for all interviews.*

*Kat: Yes, and then we went on to compare diachrony and synchrony across the interviews to get the generic structures.*

#### Derivation of generic structures

Specific structures map the diachronic and synchronic dimensions of single experiences. Generic structures put such specific structures in relation, thus allowing us to compile and compare experiences across trials, interviewees or conditions. Depending on the research question, this might, for example, allow for an overview of possible dimensions of a certain experience type or allow mapping of individual differences.

To exemplify the generation of the full diachronic generic structure of our data would require reporting on all five interviews and their experiences, which exceeds the scope of this paper. Instead, this sub-section presents the outcome of this diachronic comparison and then illustrates the genesis of a synchronic generic structure of remembering a successful interview. This is exemplified by bringing in specific structures from one moment in Christian Suhr's experience and one in Martijn van Beek's, both of whom described a sense of connection (Christian) or contact (Martijn) as a pivotal part of remembering the successful interview.^20^

**Fig. 3:**
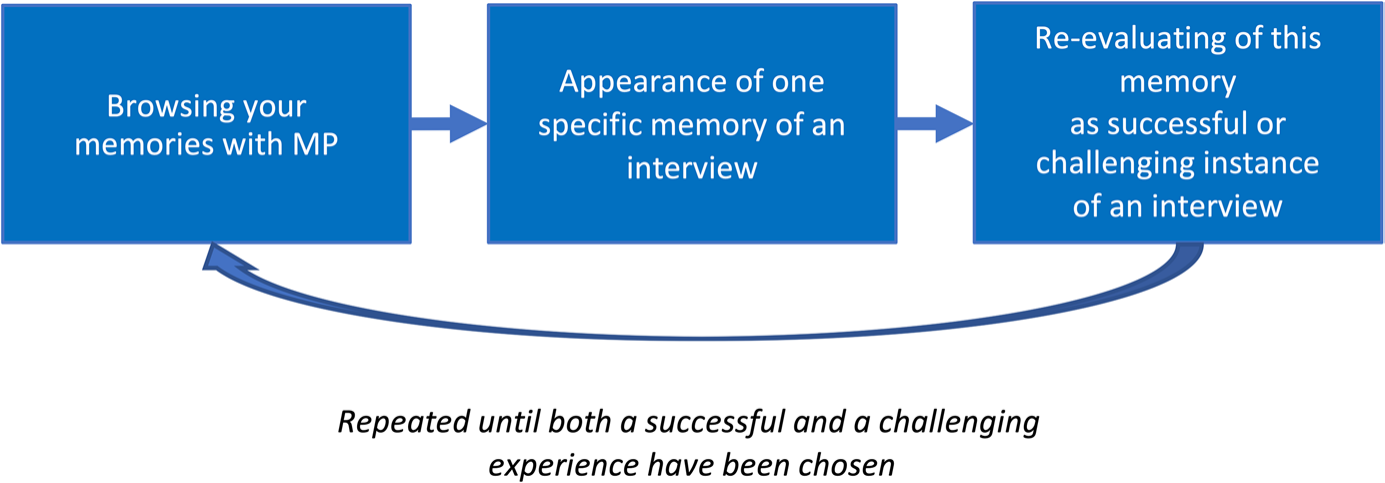
Generic diachronic structure of the experience of searching or browsing your memory for one successful and one challenging instance of micro-phenomenological interviewing

*Kat: Let's start with sharing the generic diachrony that we derived from all the interviews of this pilot as illustrated in *Fig. 3*:*

*1) The experiences of all participants started with a search, as an open stage of browsing through a large landscape of memories.*

*2) At a certain moment a distinct memory rises and manifests into consciousness as either fitting or not fitting the task.*

*3) In the experiences of three of the five interviewees, this selection is re-evaluated by a more thorough and active assessment of the experience performed. This might happen, either immediately after the experience is remembered (valid for two interviewees, in both conditions), or at the very end of the selection process (valid for one interviewee).*

*Phases 1, 2, and eventually 3 are then repeated until an instance of each condition (successful or challenging) is chosen for presentation to the interviewer﻿.*

*Bess: So, this generic diachrony ignores the specifics of each individual experience for the purpose of pointing out their common temporal structure. For example, here it is not mentioned that I first recalled a positive experience, quickly and spontaneously–the interview with Leonore. Instead, it says "appearance of one specific memory of an interview"–this leaves open how fast or effortful it appeared and whether it was a positive or challenging interview.*

*Kat: Indeed. Generic structures (synchronic and diachronic) can be of two types, depending on experimental design and your research question. They may give an overview of all the dimensions of a type of experience that are suggested by the data, thus comprising all those categories and instances that may only be expressed by one interviewee. We will describe such a structure later in the synchronic section. Alternatively, generic structures may describe the common characteristics only: what is shared across all interviews. In this sense, *Fig. 3* illustrates a very rough diachronic structure that all experiences shared, but generally speaking, derivation of generic structures focus on what is shared or coherent between interviews.*^21^

*Bess: Maybe it should be said here that, although every experience is in some sense unique, coherence and repeatability validates micro-phenomenological research. This plays out in at least four ways. Firstly, on the level of a single interview: If, despite the iterative structure of the interview, the consistency of the interviewee’s report is high, this indicates reliable data. Secondly, on the level of the generic analysis: the recurrence of structures across experiences reported by different interviewees or over interviews suggests reliability.*^22^* Thirdly, on the inter-researcher level: when different researchers repeat the entire study, or when different analysts are given the same raw data, and find similar structures, this indicates reliability through replicability. Fourthly, the validation of results may come from triangulation between different research methods. When a different research method is applied, do the structures identified correspond to other findings? For example, when micro-phenomenology is combined with neuroimaging *(Lutz, 2002; Lutz et al., 2002; Petitmengin & Lachaux, 2013; Varela, 1996)*, the detection of the neural correlates of an experiential structure is a mutual confirmation of the validity of both findings.*

*Kat: There may even be a fifth way: Sometimes, micro-phenomenological interviews and their analyses have been shown to inform practices. For example, micro-phenomenology has been used in the teaching of fashion design *(Petreca, 2016)*, and seizure preventive strategies for epilepsy have been developed using micro-phenomenology *(Petitmengin et al., 2006)*. But regarding this study and our current concern with the derivation of generic structures, we are dealing with the second type of validation: the recurrence of structures across experiences reported by different interviewees.*

*Bess: Shall we then move on to how we derive generic synchronic dimensions?*

*Kat: For this, let's go back to this "sense of connecting" that you experienced in the re-evaluation phase of your experience. Early on, I was struck by an unexpected consistency across interviews. For example, all interviewees described a bodily aspect as one of the validation criteria for their choice of a successful interview.*

*Bess: Right, let's have a closer look at this recurring bodily aspect.*

*Kat: In your case, two moments spontaneously popped up along with a "sense of connecting". For at least one of these moments, this sense was experienced visually and bodily. Your descriptions led us to derive a number of further synchronic characteristics for both of these modalities. To sum up *Fig. 2*: Visually, you experience Leonore walking in front of you, you see the scene from a first-person perspective. By bodily, you refer to a sensation that you then specify further in a number of sub-categories, for instance: It's being in the upper and lower stomach (bodily location). I was quite surprised that, in all the other reports about remembering a successful instance of micro-phenomenology, I identified quite similar descriptive categories.*

*Let's have a look at the descriptemes of Christian's successful interview during his searching phase. His interviewee is called Marc.*

*"Marc's figure … this shape is kind of covering around half of his body. […] So, he's like a fuzzy presence behind. It's kind of dark, I can't see his eyes or something, just the contours. […] there is a sensation in my chest and around my heart as well. [laughs][…] So it's like a little… I don't know how to express… not a vibration… but the feeling of… a kind of touch. […] Yeah, you could almost have the same sensation by doing this [repeatedly puts gentle but firm pressure on his chest with his right flat hand]. […] from the chest region which… feels like a connection, you could say… some kind of attentive connection that kind of flows here [moves his hand from his chest towards me and back] […]. And when I say connection to Marc or to [that which] he was describing – it's not a cord, not like that. It's just… very faint. Subtle. […] There's a flow in both directions."*

**Fig. 4:**
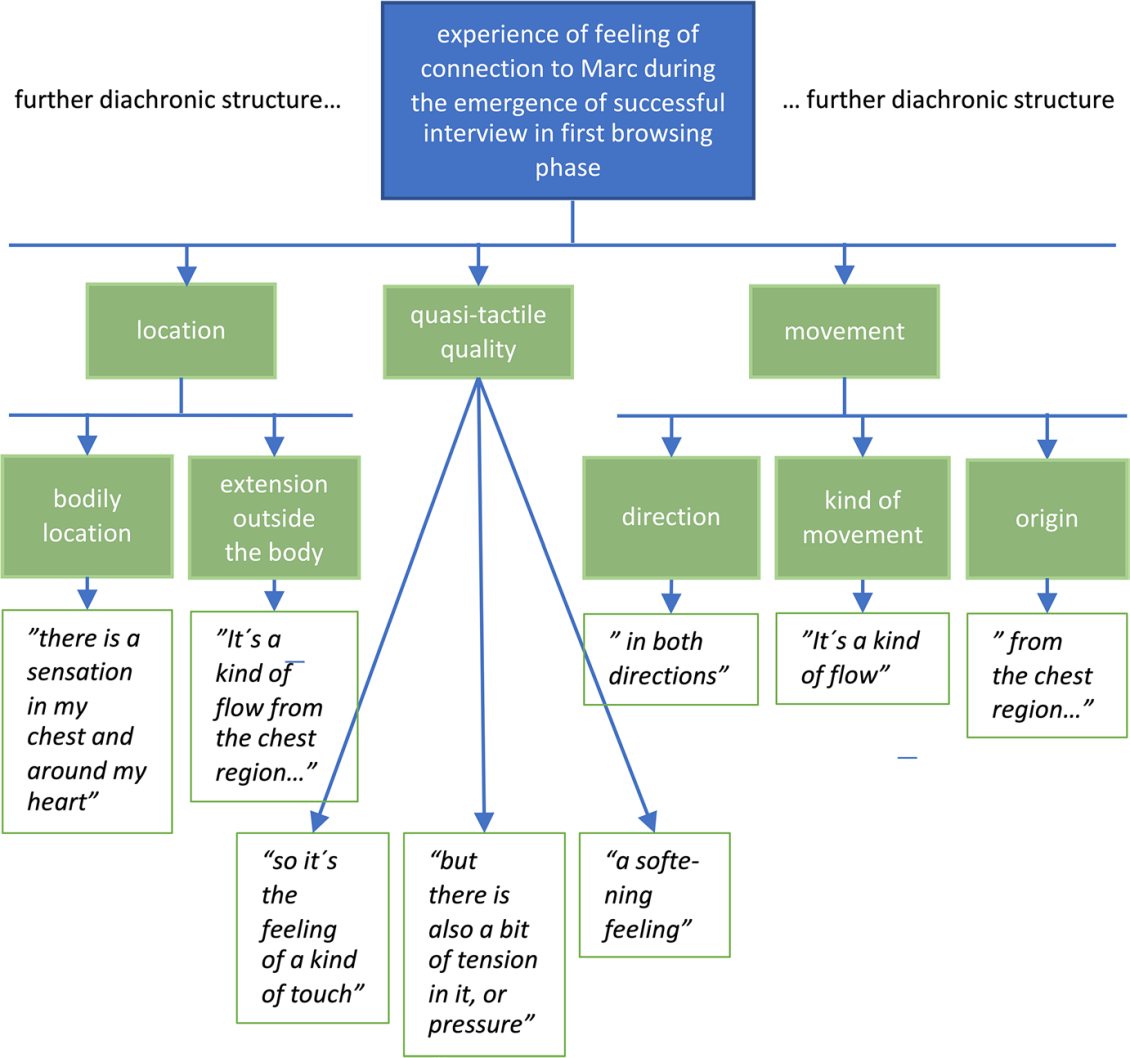
The figure shows the specific synchronic structures of Christian´s first memory experiences of the positive interview

*Bess: There are quite obviously similarities to my experience: Firstly, of course, he reports about "feeling a connection". I was talking about the "sense of connecting"–this seems very close. There is also a visual part of him seeing the interviewee and imagining something the interviewee talked about. And there was the "bodily" part, this sensation seems to have a location similar to the one I report as it is captured by *Fig. 4

*Hearing his description, even if similar to mine, I wonder whether a reader not familiar with describing subjective experience might raise an eyebrow at these words. They might sound a bit esoteric or ungrounded?*

*Kat: Well, in a micro-phenomenological interview we are asked to describe what we live through, our experience. This is often quite hard to fit into words and there is, of course, always risk that we are dealing with conceptual metaphors or that an interviewee confabulates part of an experience. However, it is important that a description must not be discredited just for the reason of it sounding unfamiliar. In particular, Chris´ report contains examples of what is called transmodal experiences (see also *Petitmengin, 2007*). Such experiences do not fall within a specific sensory modality and are therefore often even harder to fit into words and common categories: strictly speaking, they are not images, kinaesthetic, tactile sensations, or sounds, but are experiences that cross the borders of these categories: They often have specific sub-modalities such as intensity, direction, movement and rhythm that can describe different sensory registers. And while, as with anything we experience, these sensations are likely influenced by cultural embedding, they need not be confabulations or artefacts of a conceptual discourse.*

*Bess: Earlier, you mentioned that practice can have an influence on your ability to articulate experience, which reminded me of Martijn van Beek's interview.*

*Kat: Let's take a close look: Here is Martijn's description of how the memory of the successful interview comes up in the initial search:*

"*The image of the interview, of the situation, of the person. […] Yeah, I see that person, and the room, totally the way it was. […] A visual image… and then the memory of the contact between us […] I can actually sense the contact. It's a ... it's not easy to put into words, but there's a sense of ... of ease, trust, sort of this kind of atmosphere. And then it's… physically all centred around the heart, sort of warm, intense. Like it's related to practice, it immediately goes into this practice landscape. […] Because... it opens up what opens up when I practice my meditation*."

**Fig. 5:**
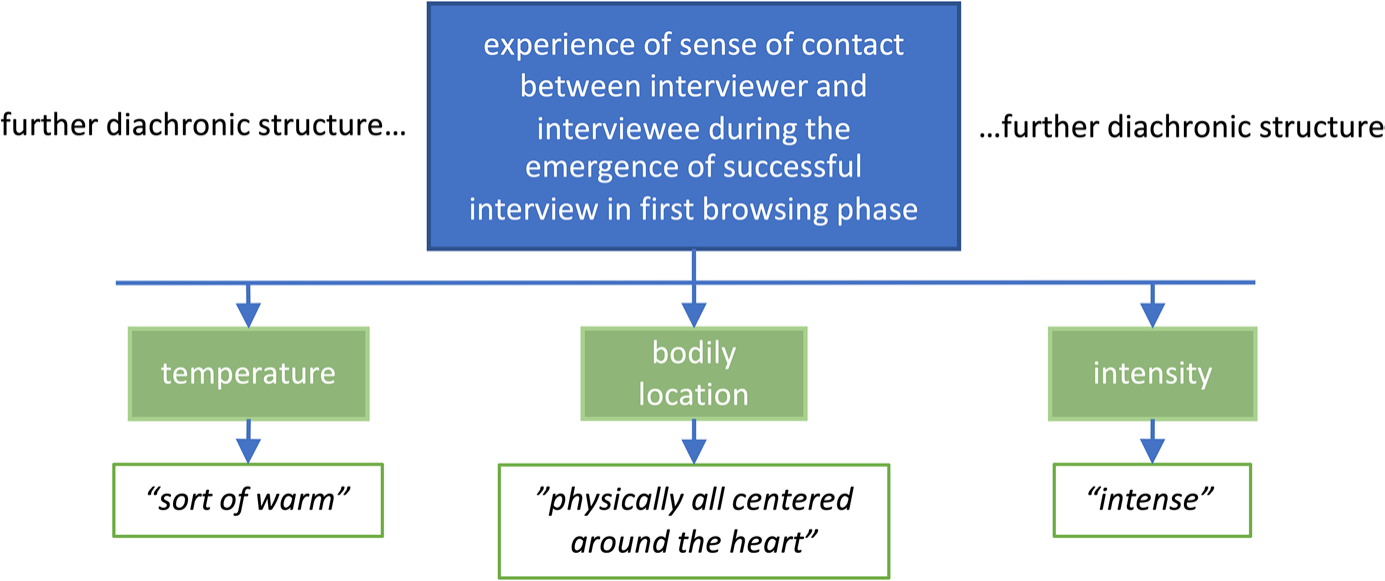
The figure shows the specific synchronic structure of Martijn´s first memory experiences of the positive interview

*5Bess: Some of this sounds similar to my experience again. He is sensing a special "contact", as he calls it, physically located around the heart, warm in temperature, etc. – see the abstractions in *Fig. 5*.*

**Fig. 6:**
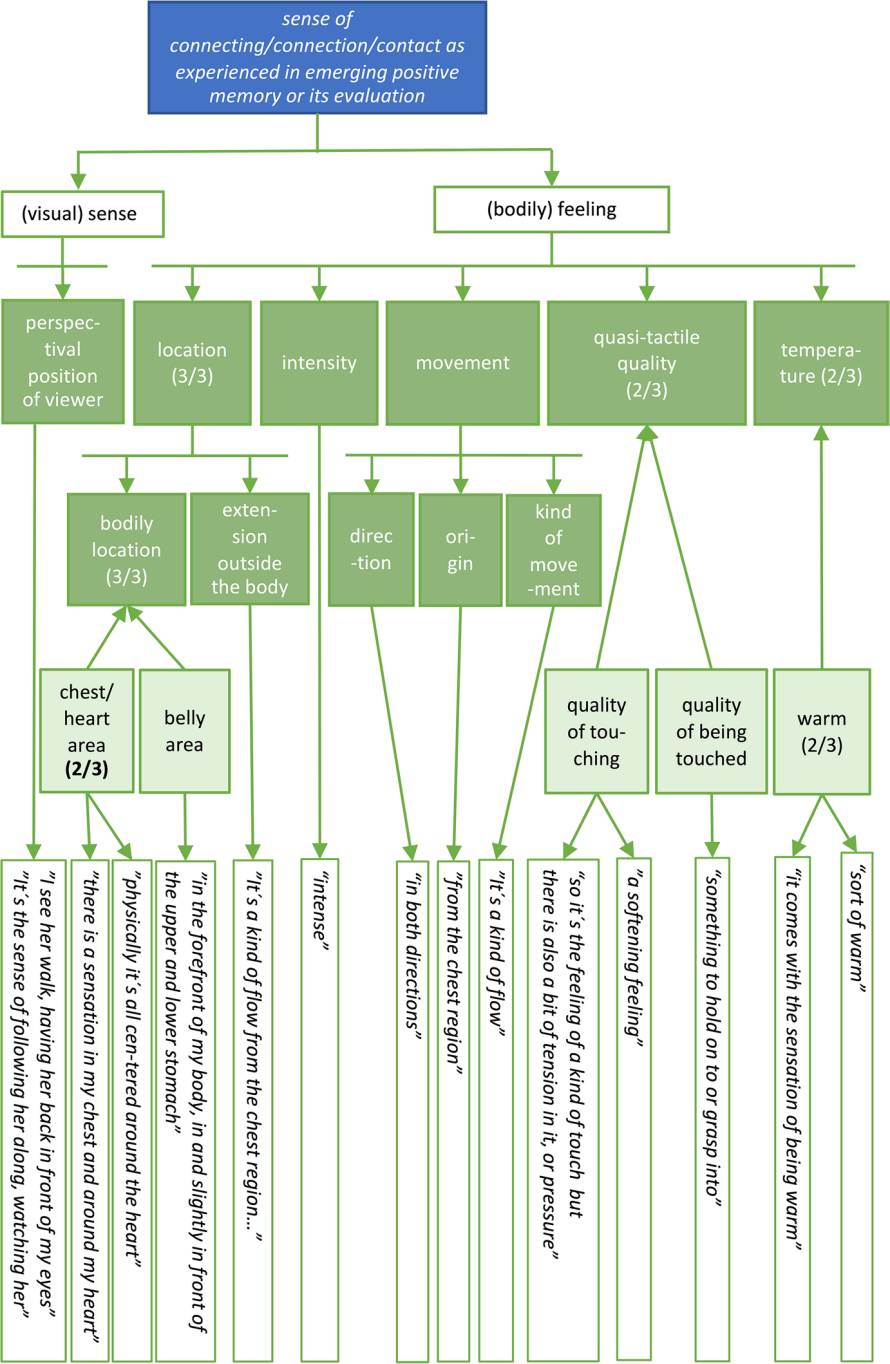
The generic structure of the "sense/feeling of connecting/connection/contact". The numbers indicate how many interviews (x out of 3) showed instances of the respective category. They are added only for x > 1

*Bess: What about the sense of "ease and trust" he mentions?*

*Kat: This is an example of not detailed enough data. I should have asked more questions to know how precisely Martijn experienced this. I actually attempted to do so in the interview, but the report drifted into more general descriptions of meditation practice.*

*Bess: Yes, when touching upon an expertise of theirs, even highly trained interviewees often tend to speak more generally rather than referring to a singular experience. This can be very informative but does not deliver experiential data.*

*Kat: Right. I still feel we have enough data to exemplify how to construct a generic synchronic structure. Though, as we are dealing with a very limited sample here, with data from a few interviews, the generic synchronic structure can not only focus on recurrent features. Instead, Fig. **6** gives an overview of all dimensions this type of experience contains, as suggested by the data. So, it can show similarities and consistency, but also maps out unique dimensions.*

*Bess: For example, not all interviewees described the target sensation as having a temperature: Still, this category is part of the generic synchronic structure as portrayed in Fig. **4**, where numbers indicate how many of the specific structures showed evidence of this dimension. It's worth highlighting that this figure covers several dimensions, the derivation of which has not been described in this article. And the boxes in lighter green represent specialised categories that allow further comparison.*

*Kat: Yes, so the analysis first focuses on abstracting the structural categories involved in a certain experience type. In this example, the sense of connecting is characterised as having a bodily location. Using specialised categories, we can then refine the structural categories initially identified, for example, by highlighting different possible locations, such as the belly and the chest. This better allows us to compare instances within and across participants: for example, how many interviewees allocated the sensation in one more specific area, like the chest/heart.*

*Bess: Thus, Fig. **6** is a result of the micro-phenomenological analysis.*

*Kat: Yes, you could say that, taking into account that this is an analysis of only part of the data of a small pilot study.*

*Bess: Shall we then sum up and discuss? What do we think about these data now? And how do we plan to work with this in the future? Kat: Let´s do that.*

## Summary and discussion

*Kat: So, first, the main purpose here was to introduce the method of micro-phenomenology by offering a lived impression of its procedures. For this, we ran five micro-phenomenological interviews, one of which we then condensed and edited as an example of the method. The target experience of these interviews was a short memory task that required the interviewees – all researchers experienced with mico-pheomenology – to browse their memory for one successful and one challenging interview. We hoped to elucidate what makes for successes and failures with the method, as well as provide a demonstration of the method's capacities.*

*Bess: Yes. And the experiences explored indicate that success might often be associated with a sense of connection or contact between them and the interviewee, often involving a bodily sensation. This aspect really differs from what we thought before the investigation, just based on talking with researchers about what makes for a good interview.*

*Kat: Exactly. Usually, what comes up in these conversations is the importance of external circumstances – such as a good experimental design, choosing a specific experience of distinct and short duration. As well as the natural or trained abilities of interviewees to describe their experience, and the skill of the interviewer in accompanying and guiding them without inducing anything themselves.*

*Bess: But these are important, too, right?*

*Kat: Yes. Especially as they need to be considered when selecting micro-phenomenology amongst alternative research methods. But in all our interviews, the first thing that came up as a marker of successful interviews was a sense of connecting, connection or contact, thus something about the quality of the interaction between interviewee and interviewer. This suggests that relationality is a key to successful micro-phenomenological interviews and is an area to follow up beyond this pilot.*

*Bess: Does that mean that if this ‘sense of connecting/connection or contact’ is not felt or remembered, the interview necessarily lacks quality?*

*Kat: No. Firstly, it is important to keep in mind that the interviews covered here assessed the target experience of browsing one's memory for a successful interview and not an interview experience itself. Therefore, it is not given that this sense, especially in the detailed synchronic characteristics outlined here, is experienced while actually doing the interview. But even if we would find similar structures in a wider data set, the most we could say is that it might be an indicator of a successful interview–not a necessary characteristic.*

*Bess: But then, what can we gain from an understanding that relationality seems central here? Is it important for micro-phenomenology in general?*

*Kat: I think so! We present evidence that the remembered connection between interviewer and interviewee is experienced to influence the perceived quality of micro-phenomenological interviews, maybe it helps to evoke and articulate experience. Relationality has been noted before as being of fundamental importance in fields of qualitative research, such as in the feminist tradition, where qualities such as attentive listening and responsiveness are seen as key *(Gilligan, 1982; Oakley, 2016; Tronto, 1993)*. And especially in terms of micro-phenomenology there seems more to know and confirm here. So, what this really calls for is further research on this matter!*

*Bess: And what precisely would be advisable to do? Extend our sample to turn this pilot into a more complete study?*

*Kat: Yes, a first step would be to conduct more interviews, ideally until the reports we obtain do not suggest additional new categories, or values. When we keep getting the same categories repeatedly, this is known as ‘categorical saturation’ and is a good indication of reliability.*

*Bess: I guess it could also be important to investigate whether a corresponding experience exists on the interviewee's side and how the challenging interview experience is remembered.*

*Kat: Indeed. In this pilot, contributors just happened to select instances where they were interviewers. It would be interesting to see if the "contact" was felt on both sides. But most important I would deem to explore better how this contact gets established, what breaks or prevents it and what about it is specific to micro-phenomenological interviews with its particular rules such as the literal repetition of words and gestures. In this respect,it also seems essential to compare our findings and inform further steps through related findings in sociology, psychology and psychotherapy. For example, *Fonagy and Bateman (2012)* have stressed that mirroring and fine-grained attunement of speech and gestures plays a fundamental role in mother-child and patient-therapist relationships. Daniel Stern described transmodal attunement as a fundamental way of creating meaning throughout the entire lifespan *(Stern, 1985, 2010).* Finally, *Oakley (2016)* pointed out that the bonds between interviewer and interviewed established in these ways are particularly complex and under-explored and clearly need further investitation to enhance our understanding and design of qualitative methods..*

*Bess: Whee! Loads of roads to go. I look forward to that.*

**Final remarks**

This article illustrated the micro-phenomenological interview and analysis technique via a pilot study and an auto-ethnographic description. Over the course of this pilot, the interview and analysis focused on a *sense of connection* that the interviewers used to identify the quality of a remembered interview. Overall, we hope to have demonstrated that micro-phenomenology is well suited to help interviewees describe subtle aspects and micro-dynamics of experience that they might not otherwise spontaneously attend to or articulate and to compare such describtions across interview and participants to identify differences as well as generic structures.

Practically speaking, the method is suitable for use with naive participants. However, it needs to be carefully introduced–ideally with a practice interview. Data quality can be significantly enhanced when it involves trained and experienced interviewees, as they will have developed their capacity to describe experiences. Also, training in meditation, mindfulness, and focusing, are thought to enable more detailed and articulate reports, possibly due to enhanced skills in attending and articulating experience (Petitmengin et al., 2017). Trained and ideally experienced interviewers are requisite as interviewers need to build their capacity to guide the interviewee in the evocation state and to explore diachronic and synchronic dimensions of the experience through reformulation and non-inductive (open) questions.

When it comes to contrasting micro-phenomenology to other methods, micro-phenomenology is quite flexible concerning experimental design. As described, micro-phenomenology is applicable to many research areas and has influenced fields as diverse as clinical and psychological neuroscience (Lutz & Thompson, 2003; Lutz et al., 2002; Petitmengin et al., 2006) to textile and fashion design (Petreca, 2016). The method can be applied to describe past experiences of everyday life and experiences elicited immediately before an interview in an experimental setup. This flexibility distinguishes it, for example, from Descriptive Experience Sampling (DES), which insists on a lab-based random sampling technique (Krumm and Hurlburt, this issue). However, similar to DES, micro-phenomenology interviews and analyses depend on the selection of singular experiences: for example, the tasting of a Bordeaux wine last night as opposed to the tasting of wine in general. Also, micro-phenomenology is best suited to investigate short instances as the fine-grained exploration of an experience of a few seconds often takes considerable time to explore, making it harder to apply to more open research questions. In these cases, other techniques such as Interpretative Phenomenological Analysis (Smith et al., 2009) may be more appropriate. When the research question cannot be tackled by relying only on specific instances of one experience, it is also possible to combine micro-phenomenology with other methods, such as the Biographical Narrative Interview (Depraz, 2021). For a closer comparison of micro-Phenomenology with Hurlburt's DES method, see Petitmengin (2011), and for comparisons to the Descriptive Phenomenological Psychological Method, Interpretative Phenomenological Analysis and Grounded Theory, see Petitmengin et al. (2018). For a comparison with Høffding and Martiny's Phenomenological Interview, see Bitbol (2018).

By its very name, micro-phenomenology implies a close relation phenomenology–a suggestion that has been the subject of extensive debate (Petitmengin, 2009; Petitmengin & Bitbol, 2009; Zahavi, 2007 and 2012; Gallagher, 2003). In a nutshell, we hold that micro-phenomenology (as a tool that involves experimental design, interviews and analyses) shares some questions and goals with phenomenology in at least two ways.

First, both phenomenology and most micro-phenomenological studies aim to achieve something more than introspective reports of singular events. They are both interested in detecting generic structures of a type of experience. While currently not all micro-phenomenology results are embedded in a transcendental discourse and discussed in the context of the existing phenomenological work, they have the potential to be. It has been pointed out before, by phenomenologists, that use of micro-phenomenology to investigate the existence of generic structures of types of experiences has great potential, especially in the field of neuro-phenomenology (Gallagher, 2003).

Second, micro-phenomenology is preoccupied with Husserl’s intention “Die noch stumme Erfahrung zur reinen Aussprache ihres eigenen Sinns zu bringen” (“To bring the as yet silent experience to purely express its own meaning.” (Husserl, 1950, p. 77)). Articulation of experience, however, as required by micro-phenomenology as well as phenomenology, is an intervention. Therefore, both methods share the question of what changes are incurred due to the research process undertaken (Petitmengin & Bitbol, 2013; see Petitmengin & Bitbol, 2009, pp. 387–390). In this context, it is worth mentioning that, previously, it has been proposed that through micro-phenomenology we can come in contact with ‘pre-reflective’ experiences. Based on discussions, see Zahavi (2011) and Petitmengin (2010), we no longer use this terminology. While the full discussion cannot be portrayed in this paper, it should at least be said that the term ‘pre-reflective’, in the phenomenological literature, has been reserved  to refer to what “it is like” to (simply) live something, without reflection. This can involve structural dimensions of experiences such as minimal self-awareness, bodily self-awareness or internal time consciousness, or simply un-investigated experiences. Micro-phenomenology, however, does not facilitate the full recovery of such an original experience but rather a re-enactment of it in which participants are guided to, attend to and articulate previously at least partly ‘unnoticed’ or ‘unrecognised’ elements and aspects of the target experience. Asmentioned above, the method relies on the observation that, most of the time, our attention is allocated to the superficial "what" and the “why” of experience, which can conceal the more fine-grained "how". Particular expertise, or help from an expert interviewer, is needed to reorient and let such initially unnoticed elements of the experience emerge into awareness. The interviewer's expertise thus consists of deliberate perlocutionary acts, designed to trigger the interviewee to evoke the experience and perform accurate attentional movements that will allow them to recognise initially unnoticed elements of this experience, as illustrated in the preceding dialogue. While the question of how the attended and articulated experience differs from its original target remains open, progress can be made by evaluating the reliability of both the interviewers' and the interviewees' acts. In short, micro-phenomenology advocates a "performative" view of the reliability of a description (see also Petitmengin and Bitbol (2009) and Petitmengin (2021)).

It is also worth highlighting that experiential reports are given in hindsight. Therefore, micro-phenomenology needs to take account of the known limits and pitfalls of memory report–particularly, the unwelcome possibility that interviewing could lead to confabulation (when reports are shown to diverge from what can have happened). This problem can be regarded as a cause for the earlier rejection of introspection and the rise of anti-phenomenological behaviourism. For example, Nisbett and Wilson (1977) suggested that we have no trustworthy introspective access to our cognitive processes due to confabulation, especially in experimental situations when participants might follow demand characteristics. Following Nisbett and Wilson (1977), Johannson et al. (2006) showed participants two pictures of women's faces and asked them to choose which one they found the most attractive. Immediately after, they were shown the selected picture again and asked to explain the reasons for their choice. However, in some cases, the picture was replaced by the one that the participant did not choose. The participants detected the trick in only 20% of cases, while in 80% of cases, they provided an explanation for the choice they did not make. This result seems to support the hypothesis that we have very limited introspective access to our choice processes, and by extension, to all our cognitive processes.

Micro-phenomenological procedures have been developed to minimise this possibility, particularly around avoiding leading the participant. It is important to recognise that the Nisbett and Wilson confabulations arose when participants were deliberately misled and asked to speculate on reasons for choice – *why* questions. Micro-phenomenological interviews instead gently invite the interviewees to remember and report about a specific point in time, without speculation and with open questions, such as *how?* Also, the interviewers are strongly advised against using leading questions or paraphrasing.

In 2013, the Nisbett and Wilson's experiment was reproduced with the adjunct of micro-phenomenology (Petitmengin et al., 2013). A micro-phenomenological interview was conducted in which the participants were helped to describe their choice process through neutral and precise questions by a skilled interviewer, before being shown the wrong picture. In the trials where participants did not undergo the interview, the results were similar to those of Nisbett and Wilson, and the participants only gave scant responses regarding their choice processes. However, in the trials in which they underwent a micro-phenomenological interview on their choice, their explanations were more in-depth and importantly they detected the trick in 80% of cases. This can be interpreted as reversing the classic Nisbett and Wilson finding and showing the potential of micro-phenomenology as a powerful tool to become aware of and articulate subjective experience.

In this vein, we suggest that micro-phenomenology presents a rigorous method that allows us to probe experience and hope this article gives the reader an impression of what is required in, and can be achieved by, micro-phenomenological investigations.

## Footnotes

1 Such judgement and explanations are called “satellites” in the micro-phenomenological jargon. While satellites and contextual information are often an unavoidable part of interviews and might gain importance in later stages of interpretation, such data is not included in the analysis steps immediately following the data recording. See also Petitmengin (2006), Petitmengin et al. (2018), Valenzuela-Moguillansky and Vásquez-Rosati (2019).

2 Since the experiences shared in micro-phenomenological interviews can feel intimate, as they might never have been voiced before in such detail, we consider the repeated establishment of this ‘privacy contract’ to be essential. We recommend making this explicit and repeatedly reminding the interviewee of it throughout the interview, regardless of whether they are trained or untrained, known or unknown before each interview.

3 Note that Kat here has given Bess a task with the intention of creating an experience that itself became the subject of the following interview. However, it is important to keep in mind that Kat and Bess in this case are now exploring *the experience of selecting an interview* rather than *the experience of a particular interview*. This variation on selecting a target experience has been used before, especially in pilot settings, where it can help with feasibility and identify significant experiences of a specific type (see, for example, for experiences of love: Suhr et al., 2021), but has the potential to remove the investigation from its ideal target and can cause complications in knowing which monument is referred to.

4 This is a typical instance of the interviewer helping the interviewee evoke an experience: Kat suggests specific moments to which they might return. She thereby creates a set of possibilities for Bess to reconnect to her experience through what she was hearing, sensing physically, and otherwise noticing in that moment.

5 In our experience, a slight withdrawal from the current situation seems to benefit evocation and listening. For example, it can help the interviewee to close their eyes or at least to avoid unrelenting eye contact with the interviewer. Most interviewers also depend on extensive notes to assist the repetition of the interviewee’s utterances, which often naturally involves looking down instead of looking at the interviewee. Nevertheless, it is important for the interviewer not to withdraw completely into such notes, but to attend to the interviewee to support them when necessary and take note of gestures and other non-verbal expressions.

6 It is important to give the interviewee a precise moment from which to start the description. Here Bess, as an experienced micro-phenomenologist, is redefining this beginning moment by stressing that her experience of browsing started earlier than Kat expected. This might not be as easy for inexperienced interviewees. Piloting the task to get a sense of where to begin is a good way to mitigate such difficulties.

7 This exemplifies repeating the interviewee’s words back to them. The primary objective here is to get an overview of the rough timeline of the experience and to check for misunderstandings or additional sub-moments not mentioned, or yet remembered, in the first report. It is essential for this endeavour, that the interviewer, when introducing the interview technique, really encourages the interviewee to interrupt them at any time they feel like correcting or complementing the report. Similarly, it is important, that the interviewer, during the repetition, speaks slowly and adds short breaks so that the interviewee can easier step in with corrections, additional information or even, as in this case, approval.

8 Note this as a typical instance of examining if a statement actually represents experiential data, that is whether it is expressing what was lived in the moment of the target experience or whether it rather represents a “satellite”, that is an explanation of or comment on the experience in hindsight. This distinction will become crucial in the later preprocessing of the data, that deselects satellites from the further analysed material. Two cues that made Kat wonder whether she was dealing with a satellite: Firstly “being resourceful” and “used in a presentation afterwards” are quite abstract descriptions that do not seem to refer to a specific memory or similar. Secondly, Bess changed her tense while talking: she switches from a description in the past tense (“I quickly came across a positive one”) to the present tense (“It is very resourceful”)—which could indicate that this is something she is thinking in the moment of the interview rather than during the target experience. The “how-question” Kat uses (“How did you experience this aspect in this moment”) allows exploration without Kat uttering doubt, which might have unsettled the interviewee. Instead, the question allows the interviewee to elaborate on how this aspect was experienced and to help identify it as a "satellite", not part of the target experience.

9 Bess’s comment confirms that the description of “it being resourceful and used in a presentation afterwards” was likely a satellite or otherwise unreliable, so it is not further pursued or used in the subsequent stages of analyses.

10 Here Bess corrects the assumption implied in Kat’s wording and adds additional moments to the time sequence.

11 In the original interview, Kat started to explore the experience from the start, retrieving further details about the moments of browsing, the retrieval of the first memory etc. We omitted this part of the transcript for brevity.

12 Notice how Kat reiterates the privacy contract (“would you agree”) and encourages Bess to take a patient and receptive attitude (“take your time”, “let this moment come back”). Evocation is a process which cannot be forced (Petitmengin, 2006). This question also marks where Kat shifts the interview from developing general or coarse temporal structure to focusing on a specific moment.

13 All interviewees, other than authors, have been anonymized.

14 Here we see a demonstration of how some satellites are a necessary part of interviews. In the first two sentences, Bess gives the context of the experience. This means that they do not contain any actual experiential data, though they are essential for the interviewer to understand the target experience. The experiential report then starts with the imagination of a flower. Interviewees less experienced than Bess can be prone to getting lost in the description of context or other satellite data. Not only can this make it harder to differentiate the target experience, but it might also evoke other memories that can distract from the target. For a list of different types of satellites, see Petitmengin, 2006.

15 Note how Kat leaves out the contextual satellites given in the previous turn of Bess, this helps her to focus on the target experience and attend to not only what but also how it happened.

16 Note though the ease with which Bess describes her experience on a bodily level is not common. Many interviewees would likely require further assistance to reach this level of description, for instance through additional “how questions”. Experience with the method, as well as movement and awareness practices, appear to assist with the capacity to report experience in this way. See also Petitmengin et al., 2017.

17 The involvement of interviewees in the analysis is not a standard part of the method. This current project is unusual in that the researchers Kat interviewed are also co-authors and contributed to the analysis. Exploreing such interactive processes of validation is an important area for future research.

18 The micro-phenomenological analysis technique is described at length in Petitmengin et al., 2018 and Valenzuela-Moguillansky and Vásquez-Rosati, 2019. For other ways of analysing data gained from micro-phenomenological interviews, see Depraz et al., 2017 and Vion-Dury and Mougin 2020. Depending on the research question and goal, of course, also other qualitative analysis techniques can be applied.

19 In figures, such operations are indicated by convention via specific types of arrows: down-pointing straight arrows indicate classification/instantiation, down-pointing rectangular arrows indicate aggregation/fragmentation and up-pointing straight arrows indicate generalisation/specialisation. See Petitmengin et al., 2018. It is beyond the scope of this article to introduce the theory behind each of the operation types, but the analysis steps portrayed gives examples that should make the principle intuitively understandable.

20 In the interview with Chris Allen, this dimension was present but only superficially explored. In the interview with Claire Petitmengin we mainly focused on the experience of remembering a not successful instance of an interview.

21 Figure 3 represents one of the most minimal diachrony structures possible. For example, all of the interviews reported that a positive memory showed up first, so this could have been included to increase precision. How much detail is included in a structure depends to some extent to the research question and the goal of the investigation.

22 We have already taken steps toward replication of the study: On the 30 October 2020, participants of the Micro-phenomenology research group interviewed each other using the same paradigm as described here. While the results of these interviews are not included in this article, they suggest that the outcomes described here are valid. We hope to elaborate on this in subsequent publications.
